# The risk of developing severe bronchopulmonary dysplasia: evaluating associations with the nucleated red blood cell count at birth and volume of transfusions received

**DOI:** 10.3389/fped.2026.1880763

**Published:** 2026-08-06

**Authors:** Bailey B. Zeiler, Timothy M. Bahr, Khanh V. Lai, Joel L. Addams, Elizabeth F. Stone, Erica A. Swenson, Robin K. Ohls, Robert D. Christensen

**Affiliations:** 1 Division of Neonatology, Department of Pediatrics, University of Utah, Salt Lake City, UT, United States; 2 Neonatology Research and Clinical Program, Intermountain Health, Murray, UT, United States; 3 Division of Pulmonology & Sleep Medicine, Department of Pediatrics, University of Utah, Salt Lake City, UT, United States; 4 Department of Pathology, University of Utah Health, and ARUP Laboratories, Salt Lake City, UT, United States; 5 Transfusion Medicine, Department of Pathology and Cell Biology, Columbia University Irving Medical Center, New York, NY, United States

**Keywords:** adult blood donors, bronchopulmonary dysplasia, nucleated red blood cells, pathogenesis, platelets, red blood cells, transfusion, umbilical cord blood

## Abstract

**Introduction:**

The nucleated red blood cell (NRBC) count at birth and blood transfusions received prior to 36 weeks postmenstrual age (PMA) are both associated with the risk of developing retinopathy of prematurity (ROP). We examined the association of these two factors with the risk of developing bronchopulmonary dysplasia (BPD).

**Methods:**

We retrospectively analyzed records of infants in five Intermountain Health Neonatal Intensive Care Units during the past four years who were born <32 weeks gestation, had an NRBC count at birth, and BPD scoring. We expressed NRBC counts as multiples of the control mean (MoM), with the control mean determined by the *refineR* algorithm. We report red blood cell (RBC) and platelet transfusions received before 36 weeks PMA.

**Results:**

The NRBC count of 896 infants was not independently associated with BPD risk. However, RBC transfusions received before 36 weeks were associated with BPD risk (*p* < 0.01). All 39 infants who developed severe BPD received multiple RBC transfusions (volume range 36–501 mL/kg) and 15 (38%) also received platelet transfusions (range 16–489 mL/kg).

**Conclusions:**

An elevated NRBC count at birth is not an independent risk factor for developing severe BPD, but the number and volume of RBC transfusions received are associated factors.

## 1 Introduction

Bronchopulmonary dysplasia (BPD) is one of the most common severe morbidities of infants born prematurely (1). Identifying newborns who are at greatest risk of developing BPD is an area of current investigation. Techniques for making those predictions include machine learning algorithms (2) and risk estimators (3). One factor that increases the risk of developing retinopathy of prematurity (ROP), another common severe morbidity of premature infants, is a high NRBC count at birth (4–7). Specifically, higher NRBC counts are associated with higher ROP risk, likely because high NRBC counts indicate fetal hypoxia (8, 9). An elevated NRBC count at birth might also be a prenatal risk factor for developing BPD. The NRBC count is not currently recognized as a BPD risk factor, and the NRBC count is not part of the published BPD machine learning algorithms or risk estimators (2, 3). However, if there is indeed a robust association between the NRBC count at birth and the risk for BPD, then including that factor in the prediction models might improve their performance.

Novel preventive efforts might derive from a more complete understanding of BPD pathogenesis. The current understanding of BPD pathogenesis is typically multifactorial involving prenatal factors such as preterm birth, genetics, chorioamnionitis, pregnancy-induced hypertension, and intrauterine growth restriction (1). Additionally, BPD pathogenesis typically involves postnatal inflammatory factors such as oxygen toxicity, pulmonary barotrauma and volutrauma, sepsis, presence of patent ductus arteriosus, and nutritional deficiencies (1). Several recent studies also demonstrate a strong association between blood transfusions received during neonatal intensive care unit (NICU) hospitalization and the subsequent development of BPD. These associations include both red blood cell (RBC) transfusions (10–13) and platelet transfusions (14–16). Whether the statistical associations between transfusions and BPD are *causal* or not is still under investigation; however, associations exist between transfusions and ROP, where studies indicate that *causality* is likely (17).

As a step toward obtaining new information needed to improve both BPD risk assessment and preventive strategies, we devised the present study. We utilized a large multicentered NICU database to investigate statistical associations between 1) the NRBC count at birth, and 2) the number and volume of RBC transfusions and platelet transfusions received, and the incidence and severity of BPD. We assessed both associations in the same cohort of premature neonates, born <32 weeks gestational age and cared for in a group of five NICUs with uniform care processes and transfusion guidelines (Supplementary Table 1). We accomplished this using a retrospective review of recent data from the laboratory, NICU, and blood banking records.

## 2 Materials and methods

### 2.1 Study design and population

This was a retrospective analysis of infants born <32 weeks gestational age, between January 2021 and December 2024, cared for in five Intermountain Health (IH) NICUs in Utah: McKay-Dee Hospital, Ogden; Primary Children's Hospital, Salt Lake City; Intermountain Medical Center, Murray; Utah Valley Regional Hospital, Provo; and St. George Regional Hospital, St. George. The protocol for conducting this retrospective, deidentified records review was approved by the IH Women and Newborn Scientific Review Board, and the Institutional Review Board, with a waiver of informed consent (IRB # 1051715). The database for this study overlaps that from our previous report on transfusions received and the incidence and severity of ROP (15). However, all the data reported in the present BPD study are unique to this study and do not overlap with that of the ROP study (although some of the infants are the same).

### 2.2 Data collection

We gathered data from the electronic medical record and stored data in REDCap (Research Electronic Data Capture, Nashville, Tennessee). Neonates were included in the analysis only if they had an NRBC count drawn within 48 hours of birth. The first NRBC count was used if they had more than one NRBC count obtained within 48 hours of birth.

### 2.3 NRBC counts at birth

NRBC counts drawn within the first 48 hours after birth were measured using Sysmex Hematology XE or XN analyzers (Sysmex America, Inc., Lincolnshire, IL) and IH Laboratory Services standard operating procedures. The NRBC counts were linked to deidentified patient information in the IH Data Warehouse to obtain patient age (in hours) at sample collection, and gestational age at birth. NRBC values in these patients were compared with NRBC reference intervals appropriate for gestational age (18). The median NRBC count of neonates <32 weeks gestation, on the day of birth, is 714 NRBC/µL blood. On the next day after birth, the median is 363 NRBC/µL blood.

### 2.4 Diagnosing and BPD severity grading

We defined BPD according to the criteria of Jensen et al., which is based on respiratory support at 36 weeks post menstrual age (PMA), regardless of prior or current oxygen therapy (1). BPD Severity was categorized as follows: No BPD if breathing room air without any respiratory support; Grade 1 if on <= 2 L/min nasal cannula; Grade 2 if on >2 L/min or non-invasive positive airway pressure; and Grade 3 if requiring invasive mechanical ventilation. In contrast to the Jensen criteria, the NICHD 2001 workshop definition classifies BPD severity at 36 weeks PMA based on supplemental oxygen requirements in infants who received ≥28 days of oxygen therapy. We selected the Jensen definition because of its current widespread use and because of its high positive predictive value for death or serious respiratory morbidity at 18–26 months corrected age and it has been validated in multiple independent cohorts. Additionally, the Jensen definition eliminates inter-center variability related to oxygen saturation targeting practices and is not confounded by altitude—a relevant consideration for our Utah-based NICUs.

### 2.5 RBC and platelet transfusions

All transfused RBCs and platelets were provided by the American National Red Cross and were drawn from volunteer adult donors. The RBC products were leukoreduced, stored in CPDA-1 [rarely (<5%) in Adsol, AS-1], and irradiated. Based on IH transfusion guidelines, a volume of 15-20 mL/kg was typically administered over a period of 3–4 hours (19). The apheresis platelet products were collected using single or double venous access kits according to the manufacturer's recommendations (Amicus Cell Separation Platform, Fresenius Kabi, Lake Zurich, IL) and Red Cross standard operating procedures. Irradiation was not performed; rather, donor platelets were subjected to pathogen inactivation using the Intercept system (Cerus Corp, Concord, CA) and stored in 70% platelet additive solution (PAS3, InterSol, Fresenius Kabi) and 30% donor plasma. IH NICU RBC and platelet transfusion guidelines were in place in all participating hospitals (19).

### 2.6 Statistical analysis

Summary statistics (means, standard deviations, medians, quartiles, and proportions) and basic statistical tests (chi-square test for categorical variables; Student's t-test for continuous variables when means were used, and Wilcoxon rank-sum test when medians were used) were applied for variable summaries and comparisons. We used logistic regression to estimate the relative change in odds of BPD per change in NRBC count at birth while controlling the potential confounders: gestational age and birth weight. We also utilized logistic regression to determine whether RBC and platelet transfusions modified the relationship between NRBC count at birth and BPD. Statistical analyses were performed in the R language and environment for statistical computing (R Foundation, Vienna, Austria).

## 3 Results

**Figure 1:**
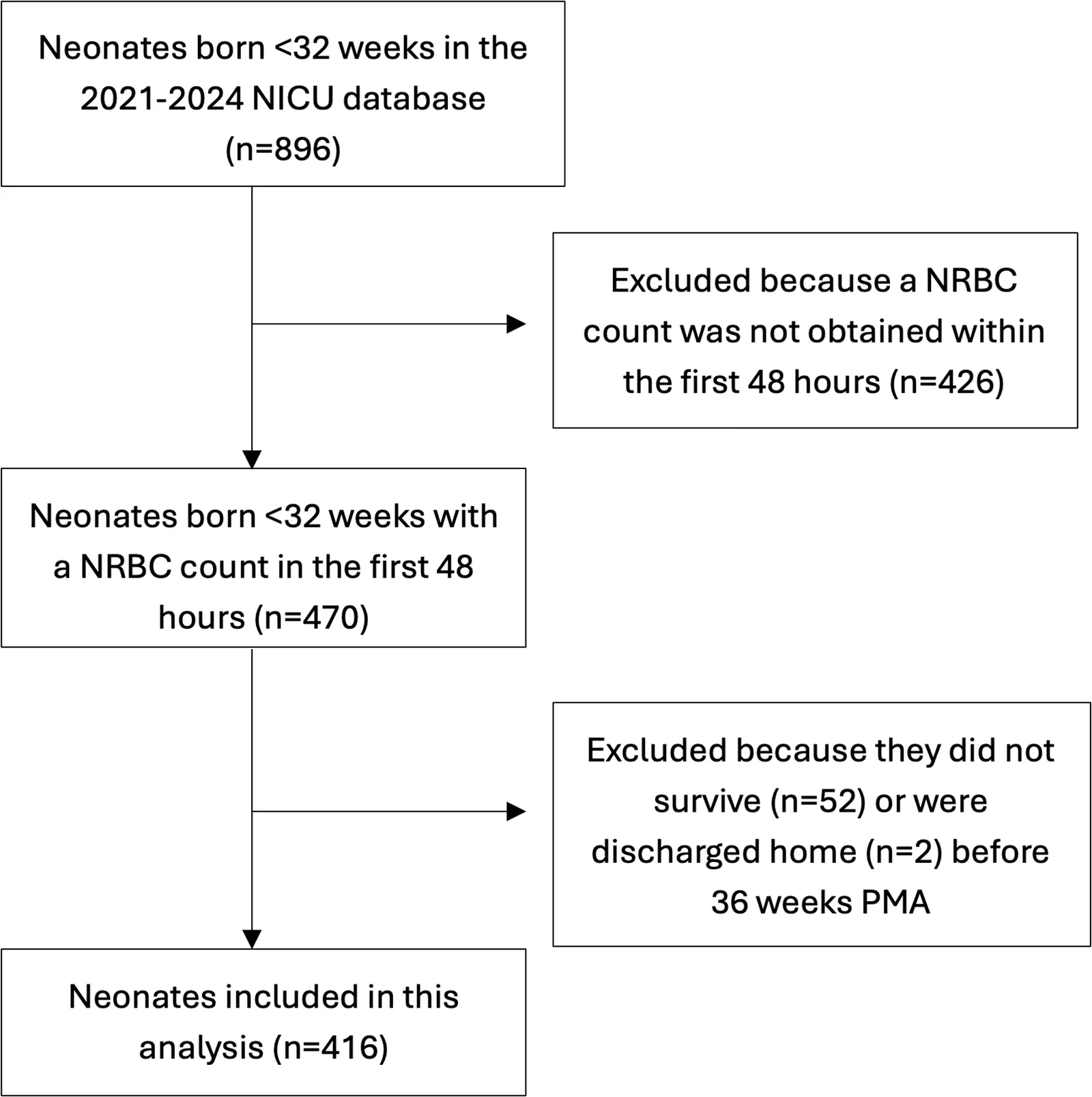
Flowchart displaying inclusion of preterm infants in this study.

Between January 1, 2021, and December 31, 2024, 896 neonates born <32 weeks gestation were cared for in the five IH NICUs. As shown in Figure 1, 470 of these neonates had an NRBC count drawn within 48 hours of birth, while 426 did not. Of the 470 who had an NRBC count drawn, 52 died, and 2 were discharged home before reaching 36 weeks PMA. Thus, 416 infants had both an NRBC count and a BPD score assigned at 36 weeks PMA and were included in the analysis.

Of the 416 infants included in the study database, 334 (80%) met BPD diagnostic criteria: 214 (66%) categorized with Grade 1; 81 (25%) with Grade 2; and 39 (9%) with Grade 3 BPD (Table 1). Infants who developed BPD had a lower birth weight and were born at an earlier gestational age than infants who did not, thus we included gestational age and birth weight as variables in the subsequent regression analyses to control for potential confounding. The five-minute Apgar score and the occurrence of IVH did not differ significantly between infants with severe (Grade 3) BPD versus those without BPD.

**Table 1:** Clinical characteristics [median (Q1, Q3) or n (%)] of the preterm infants studied who did not *vs.* did develop BPD, according to their highest BPD grade. *P-*values represent the results of Kruskal–Wallis test for continuous variables or Fisher's Exact Test for categorical variables.

	No BPD (*n* = 82)	Grade 1 BPD (*n* = 214)	Grade 2 BPD (*n* = 81)	Grade 3 BPD (*n* = 39)	*p*-value
GA (weeks)	29 (28, 30)	28 (27, 29.8)	27 (26, 29)	25 (24, 26)	<0.001
Birthweight (grams)	1283 (1086,1434)	1140 (923,1285)	895 (765,1115)	625 (558,738)	<0.001
AP 1	6 (3, 7)	5 (3, 7)	4 (2, 6)	3 (1, 4)	<0.001
AP 5	8 (7, 8)	8 (7, 8)	7 (6, 8)	7 (6, 8)	0.011
IVH					
None	66 (80.5%)	169 (79.0%)	49 (60.5%)	25 (64.1%)	0.004
Grade 1-2	13 (15.9%)	33 (15.4%)	21 (25.9%)	7 (17.9%)	
Grade ≥3	3 (3.7%)	12 (5.6%)	11 (13.6%)	7 (17.9%)	

BPD, bronchopulmonary dysplasia; GA, gestational age; BW, birth weight; AP, Apgar Score at 1 and 5 minutes; IVH, intraventricular hemorrhage.

### 3.1 The NRBC count at birth and the subsequent diagnosis of BPD

The NRBC count was higher in the group of 334 neonates who developed BPD (5.6 MoM. 95% CI; 4.5–6.6 MoM) than in the group of 82 neonates who did not (3.2 MoM; 1.8–4.8, *p* < 0.011) (18). However, after controlling for gestational age and birth weight, the NRBC count was not an independent risk factor for BPD (*p* = 0.337).

### 3.2 Transfusions and the subsequent diagnosis of BPD

Red blood cell transfusions and platelet transfusions were tabulated separately, according to the number of individual transfusions, as well as the cumulative volume of transfusions received between birth and 36 weeks PMA. As shown in Table 2, infants who did not develop BPD, or who developed Grade 1 BPD were more likely to have received no RBC transfusions and no platelet transfusions. Those who developed BPD Grade 2 or 3 had a stepwise increase in transfusions as BPD severity increased.

**Table 2:** RBC transfusions and platelet transfusions (number and volume/kg birth wt.) administered between birth and either hospital discharge or 36 weeks PMA (whichever came first) among preterm infants who did not develop BPD, *vs.* those who did develop BPD, according to their highest BPD grade. (median and 25–75 percentiles).

	No BPD (*n* = 82)	Grade 1 BPD (*n* = 214)	Grade 2 BPD (*n* = 81)	Grade 3 BPD (*n* = 39)	*p*-value
Number of RBC Tx	0 (0,0)	0 (0,0)	0 (0,1)	4 (3,8)	<0.001
Volume of RBC Tx (mL/kg)	0 (0,0)	0 (0,0)	0 (0,24)	112 (75,217)	<0.001
Number of Platelet Tx	0 (0,0)	0 (0,0)	0 (0,0)	0 (0,4)	<0.001
Volume of Platelet Tx (mL/kg)	0 (0,0)	0 (0,0)	0 (0,0)	0 (0,67)	<0.001

BPD, bronchopulmonary dysplasia; PMA, post-menstrual age; RBC, red blood cell; Tx, transfusion; *n* = number of transfusions received.

The 39 infants who developed severe (Grade 3) BPD (Supplementary Table) each had at least two RBC transfusions (median 5.8; 95% CI, 4.5–7.1), with a median total RBC transfused volume of 153 mL/kg birthweight (95% CI, 117–189). Eight of the 39 infants received their first RBC transfusion on the first day of life. The average day of life when the first RBC transfusion was received was day 10 (95% CI, 5–15). Thirty-five of the 39 infants received ≥50 mL/kg of RBCs; for those 35 neonates, the average day of life when RBC transfusions reached 50 mL/kg birth weight was day 29 (95% CI, 20–38).

In addition, 15 of the 39 infants who developed *severe* BPD received one or more platelet transfusions (median 2.5; 95% CI, 1.2–3.8), with a total cumulative platelet volume received of 86 mL/kg (95% CI, 37–135) (Supplementary Table). The average day of life when the first platelet transfusion was received was day 10 (95% CI, 5–15). Twelve of the 15 infants who received one or more platelet transfusions received a total volume of ≥50 mL/kg of platelets; for those 12 infants, the average day of life when platelet transfusions reached 50 mL/kg birth weight was day 17 (95% CI, 8–26).

## 4 Discussion

Severe BPD is diagnosed according to the Neonatal Research Network definition (1), when an infant who was born before 32 weeks gestation is receiving mechanical ventilation after reaching 36 weeks postmenstrual age (1). Successful efforts to reduce mild, moderate, and severe BPD are warranted, but reducing s*evere* BPD would produce the greatest benefits to society. This is because *severe* BPD has the most serious ramifications: it is associated with longer length of NICU hospital stay; post-NICU respiratory and neurodevelopmental morbidities and mortality; and long-term pulmonary impairments during school age, adolescence, and into adulthood (20).

One step toward reducing *severe* BPD could involve the early identification of high-risk neonates. Machine learning and risk estimators have been created for that purpose, with the goal of applying preventive strategies to infants who score as high risk (2, 3). Our present data suggests that, although an elevated NRBC count is a risk factor for developing ROP (7), it is not an independent risk factor for developing BPD. This is consistent with the finding of Marom and Mimouni et al. who in a small study sample (*n* = 39) identified no association between NRBC counts at birth and the subsequent development of BPD (21).

Novel approaches are also needed to *prevent* severe BPD. Hellstrom et al. found that neonates with severe BPD had a low fraction of fetal hemoglobin (HbF) during their first several weeks of life (22). This association between low HbF levels and high BPD incidence was recently confirmed by Dani et al. (23). Erythrocytes of infants born <32 weeks gestation have approximately 85-90% HbF and they undergo a postnatal transition from HbF to HbA synthesis that is similar to the rate of hemoglobin switchover *in utero*, with about 80% HbF by 40 weeks postnatal age and <2% HbF by six months (24). Bard and Prosmanne found that even preterm infants who underwent the stresses of NICU care, with mechanical ventilation, parenteral nutrition, and packed red cell transfusions, had the same rate of switch from HbF to HbA synthesis that occurs with normal fetuses *in utero* (24). Thus, when an infant born <32 weeks has a marked fall in HbF with a reciprocal increase in HbA [as seen in the reports of Hellstrom (22) and Dani (23)], it is because of transfusions with adult donor red blood cells, where HbF levels are typically <1% and HbA levels are 95-98%. Transfusions of adult donor RBCs are often performed to correct anemia, often resulting from serial phlebotomy for repeated laboratory testing over weeks of NICU care (14, 15).

This association between low HbF levels and high BPD incidence is consistent with the reports linking adult donor blood transfusion number with BPD incidence and severity (13–16). We found this relationship in the present cohort of preterm neonates. All 39 infants who developed severe BPD received multiple RBC transfusions before they reached 36 weeks PMA, with the average number of transfusions being 5.8. The majority received a cumulative volume of ≥50 mL/kg of RBCs. Perhaps 50 mL/kg cumulative volume of RBCs could represent a practical “red-line” risk marker for subsequent development of *severe* BPD, especially if given in the first few weeks of life.

Transfusions of adult platelets into preterm neonates are also associated, in a stepwise fashion, with BPD incidence and severity (13–16). The risk of BPD from receiving a single platelet transfusion is unclear, but our previous studies show that a high platelet transfusion burden (defined as 20 or more transfusions) virtually always predicts severe BPD or death (15). The pathogenic mechanism whereby adult donor platelets injure the neonatal lung might involve developmental differences between neonatal versus adult platelets. Neonatal platelets are relatively hyporeactive, compared with adult platelets, in response to most platelet agonists (25). Also, activated neonatal platelets express less surface P-selectin than activated adult platelets (26). Neonatal platelets may have reduced capacity to interact with neutrophils and monocytes compared with adult platelets and when preterm neonates are exposed to adult platelets, a proinflammatory milieu is fostered [25]. In our present cohort, most infants who developed severe BPD did not receive a platelet transfusion; thus, platelet transfusions are not necessary for severe BPD to occur. Nevertheless, the 39 infants with severe BPD had an average of 2.5 platelet transfusions each (range 0–16). Thus, we suspect that platelet transfusions can contribute to BPD risk severity, particularly if given in the first few weeks of life.

Our study has several limitations. First, the pathogenesis of BPD is complex, yet we focused on only two aspects, the NRBC count at birth (as a surrogate for fetal hypoxia) and the number of blood transfusions received. We control for gestational age, birth weight, mechanical ventilation status, and IVH as potential cofounders; however, other data on prenatal and postnatal inflammatory variables were not included and a limitation of this study. Thus, as a study of BPD pathogenesis, ours is fairly simplistic. However, when the present data is placed in context with the many previous and ongoing studies of BPD pathogenesis, our findings could be a relevant addition.

## 5 Conclusion

Much work remains to know whether improved transfusion practices can become an effective BPD prevention strategy. Our study did not show an association between the NRBC count and BPD development. However, our study suggests there is an association between transfusion burden and BPD severity. We judge, along with the literature, the evidence is sufficient to warrant performing the following four transfusion-reducing methods for infants born <32 weeks gestation: 1) delayed cord clamping for at least 60 seconds, or cord milking (27); 2) obtain any needed blood for initial laboratory testing using otherwise discarded umbilical cord blood, thus minimizing phlebotomy of the preterm infant (28); 3) initiate and periodically evaluate a transfusion stewardship program in the NICU, including support for restrictive transfusion guidelines, eliminating any unnecessary phlebotomies, and removing the umbilical artery catheter early (29); and 4) administer darbepoetin weekly to all neonates <1500 grams birth weight (29).

Performing these four steps will help keep the HbF levels physiologically high during the NICU course because they will reduce packed red blood cell transfusions. One additional step currently under investigation by multiple centers is, when transfusions of RBCs or platelets are needed before 36 weeks PMA, to use blood products manufactured from healthy term allogenic umbilical cord blood, rather than from adult blood donors (30). RBCs collected from umbilical cord blood are high in HbF and platelet products are hypothesized to be of the physiological hypoinflammatory fetal type. We encourage transfusion centers and perinatal centers globally to consider the potential merits of each of these BPD reduction strategies, and to engage in and support these efforts to develop transfusable products derived from umbilical cord blood.

## Data Availability

The original contributions presented in the study are included in the article/Supplementary Material, further inquiries can be directed to the corresponding author.
