# Supplementary material for: The risk of developing severe bronchopulmonary dysplasia: evaluating associations with the nucleated red blood cell count at birth and volume of transfusions received
Source: Front Pediatr. 2026 Aug 6;14:1880763. doi: 10.3389/fped.2026.1880763 (PMC13491196; doi:10.3389/fped.2026.1880763)
Supplement: Supplementary file 1 [file Datasheet1.pdf]

Supplemental Table 1: Transfusion guidelines

Product	Condition	Indication	Treatment
**LTOWB**	Massive hemorrhage	Emergency-release whole (trauma) blood	30+ mL/kg
**PRBCs**	ECMO or cyanotic heart disease	Hgb <10–12 g/dL (Hct <30–36%)	15–20 mL/kg over 2–4 hours (may be divided into 2 aliquots of 10 mL/kg each)
	Mechanical ventilation	Hgb <9 g/dL (Hct <27%)	
	CPAP with supplemental O_2; undergoing general surgery	Hgb <8 g/dL (Hct <24%)	
	Room air, absolute retic <150	Hgb <7 g/dL (Hct <21%)	
**Platelets**	ECMO, surgery, or bleeding	Platelet count <75,000/µL	10–15 mL/kg over 1–2 hours
	Before invasive procedure	Platelet count <50,000/µL	
	All others	Platelet count <25,000/µL	
