# Supplementary material for: The risk of developing severe bronchopulmonary dysplasia: evaluating associations with the nucleated red blood cell count at birth and volume of transfusions received
Source: Front Pediatr. 2026 Aug 6;14:1880763. doi: 10.3389/fped.2026.1880763 (PMC13491196; doi:10.3389/fped.2026.1880763)
Supplement: Supplementary file 2 [file Datasheet2.pdf]

Supplemental Table 2. Transfusion-related findings of 39 infants who developed *severe* BPD. Patients are listed in order of those who received the most donor RBC/kg birth weight, during their NICU stay to those who received the least.

#	GA (w/d)	NRBC/µL at birth	NRBC multiples of the mean	RBC Tx vol/kg	RBC Tx #	DOL 1st HbA	DOL 50/kg HbA	Pl Tx vol/ kg	Pl Tx #	DOL 1st Pl Tx	DOL 50/kg Pl
1	23/0	4698*	12.9	501	11	2	13	243	5	64	66
2	24/6	1580	2.2	425	18	1	5	489	14	4	20
3	25/5	667	0.9	407	13	13	21	459	13	30	31
4	23/4	1432*	3.9	377	8	1	28	0	0	-	-
5	23/6	8750	12.3	259	9	1	6	461	6	7	95
6	24/1	24198	33.9	253	16	2	3	436	16	3	5
7	23/5	1279	1.8	246	15	2	7	92	5	5	20
8	26/3	17476	24.5	229	8	1	3	297	11	2	17
9	28/5	12874*	18.0	220	10	2	14	23	1	14	-
10	24/5	9292	13.0	215	4	4	16	0	0	-	-
11	24/2	16250*	44.8	201	7	2	19	0	0	-	-
12	23/1	3608	5.1	198	11	5	20	27	1	28	-
13	25/4	9135	12.8	190	4	8	71	440	6	72	72
14	23/0	2397	3.3	181	6	3	22	0	0	-	-
15	26/4	8963	12.6	175	3	74	139	0	0	-	-
16	25/2	1396	2.0	126	6	4	13	71	3	6	36
17	23/0	4055	5.7	118	6	12	34	0	0	-	-
18	23/2	3658	5.1	114	7	1	10	63	4	1	5
19	26/4	9744	13.6	114	4	4	42	0	0	-	-
20	23/2	18182*	50.1	112	4	1	26	0	0	-	-
21	28/1	1026	1.4	100	4	19	22	174	7	19	19
22	25/0	8992	12.6	100	5	1	14	57	4	1	3
23	26/2	14280	20	100	4	5	17	0	0	-	-
24	27/5	2646	3.7	95	4	4	7	0	0	-	-
25	26/2	5768	8.1	88	3	10	37	16	1	5	-
26	23/5	1673	2.3	88	3	6	16	0	0	-	-
27	27/0	1647	2.3	85	3	6	23	0	0	-	-
28	26/1	4927	6.9	83	3	23	73	0	0	-	-
29	24/0	No CBC	-	70	4	3	41	0	0	-	-
30	24/2	8613*	23.7	66	3	12	37	0	0	-	-
31	28/5	418	0.6	60	2	88	88	0	0	-	-
32	27/6	6991	9.8	60	4	1	18	0	0	-	-
33	26/1	26453	37.0	52	2	17	42	0	0	-	-
34	30/6	1624	2.3	50	2	16	28	0	0	-	-
35	26/6	16033	22.5	49	2	9	-	0	0	-	-
36	25/2	530	0.7	44	3	3	-	0	0	-	-
37	27/0	12802	17.9	42	2	5	-	0	0	-	-
38	23/5	757	1.1	41	2	7	-	0	0	-	-
39	31/4	9189	12.9	36	2	4	-	0	0	-	-

GA, gestational age at birth; BPD, bronchopulmonary dysplasia; w/d, weeks/days; NRBC, nucleated red blood cell; RBC, red blood cell; Tx, transfusion; vol, volume; kg, kilogram; DOL, day of life; HbA, adult hemoglobin; Pl, platelet;; No CBC, no complete blood cell count was drawn during the first two days after birth. *The first CBC was drawn on day two.
